# Clocking In Time to Gate Memory Processes: The Circadian Clock Is Part of the Ins and Outs of Memory

**DOI:** 10.1155/2018/6238989

**Published:** 2018-04-12

**Authors:** Oliver Rawashdeh, Rex Parsons, Erik Maronde

**Affiliations:** ^1^School of Biomedical Sciences, The University of Queensland, Brisbane, QLD, Australia; ^2^Department of Anatomy, Goethe University Frankfurt, Frankfurt, Germany

## Abstract

Learning, memory consolidation, and retrieval are processes known to be modulated by the circadian (*circa*: about; *dies*: day) system. The circadian regulation of memory performance is evolutionarily conserved, independent of the type and complexity of the learning paradigm tested, and not specific to crepuscular, nocturnal, or diurnal organisms. In mammals, long-term memory (LTM) formation is tightly coupled to de novo gene expression of plasticity-related proteins and posttranslational modifications and relies on intact cAMP/protein kinase A (PKA)/protein kinase C (PKC)/mitogen-activated protein kinase (MAPK)/cyclic adenosine monophosphate response element-binding protein (CREB) signaling. These memory-essential signaling components cycle rhythmically in the hippocampus across the day and night and are clearly molded by an intricate interplay between the circadian system and memory. Important components of the circadian timing mechanism and its plasticity are members of the *Period* clock gene family (*Per1*, *Per2*). Interestingly, *Per1* is rhythmically expressed in mouse hippocampus. Observations suggest important and largely unexplored roles of the clock gene protein PER1 in synaptic plasticity and in the daytime-dependent modulation of learning and memory. Here, we review the latest findings on the role of the clock gene *Period 1* (*Per1*) as a candidate molecular and mechanistic blueprint for gating the daytime dependency of memory processing.

## 1. Memory Systems

### 1.1. Definition

Memory can be defined as “the retention of experience-dependent internal representations or of the capacity to reactivate or reconstruct such representations over time” [1]. “Internal representations” in the definition of memory refers to neuronal encoded representations of the surrounding environment that could guide behavior. This definition of memory fits well with the many levels of neuroscientific research [1–4] and originates from the view that all memories regardless of species and task are biological internal representations [4]. We have to bear in mind, however, that “internal representations” could also be innate constructs encoded by genes and established by developmental programs not necessarily related to learning [4].

### 1.2. History

“Memory” was initially viewed as a simple, unitary faculty of the mind and brain [5]. Later, converging evidence emerged from different scientific fields that suggested the existence of multiple dissociable memory systems [6–8]. This, however, does not mean that different memory systems can be separated according to conventional distinctions between stimulus modalities (visual or auditory) and response modalities (manual or verbal), but rather refers to different neural substrates. Evidence for the existence of multiple memory systems emerged from observations showing that different forms of brain damage in humans or experimentally imposed lesions to specific brain areas can severely impair specific forms of memory without affecting others [5, 9]. For example, lesions to the caudate nucleus or to the temporal stem connecting the inferior temporal cortex to other brain structures, among which is the caudate nucleus, impairs acquisition in monkeys trained for a long-term visual discrimination task [10–12] without influencing the acquisition of other learning tasks, like the delayed non-match to sample task (DNMS) [12]. Lesions to the hippocampus/amygdala disrupt acquisition for DNMS but not acquisition for the long-term visual discrimination task [12–14]. The information gathered from lesion experiments was consolidated into a representative map to visualize the idea of multiple memory systems and to illustrate the neural substrates involved in memory processing and storage.

### 1.3. Short-Term and Long-Term Memory

Memories can also be defined in terms of the time elapsed since information encoding. Accordingly, memories are divided into short-term and long-term memories. In general, the memory that persists for only a short period and is directly accessible after the learning experience took place is known as short-term memory (STM). Short-term memory is a labile form of memory in the context of time and is sensitive to disruption through disturbances like electroconvulsive shocks [15]. Memories that appear at a later stage after learning and last for a long time are known as long-term memories (LTM). Long-term memories are more stable forms of memories.

Psychologists have come to distinguish between STM and LTM in reference to consciousness (declarative), without focusing too much on the time lapse between information encoding and retrieval. Psychologist William James (1890) argued that primary memory or short-term memory can exist indefinitely as long as one's attention remains focused on a specific given piece of information (continuous rehearsal). However, once the information leaves consciousness (rehearsal is interrupted), even if retrieval occurs seconds later, the retrieval of that information will now involve a secondary memory system or long-term memory system.

## 2. The Circadian System and Learning and Memory

In 1885, Ebbinghaus discovered the exponential nature of forgetting, also known today as the retention curve or forgetting curve, depending on how the data is presented. In general, the retention curve presents a decline over time in the retention of what was learned [16]. In 1957, Kamin presented a retention curve for avoidance learning using rats [17]. However, the retention curve Kamin plotted was somewhat different from Ebbinghaus's retention curve. Kamin's retention curve was “U”-shaped with a minimum retention score at one hour after training. At 6 h and 24 h posttraining, the animal's memory performance was similar to the original performance measured at 1 min posttraining. Kamin postulated that one possibility to explain the “U”-shaped curvilinear function was to assume the existence of two memory systems: one memory system that dominates retention immediately after training and loses strength over time (short-term memory or primary memory system) and a secondary memory system that starts to dominate at some later stage following training, probably after some time of consolidation, and increases in strength over time. This secondary memory system is represented in Kamin's retention curve as the second rise in the “U”-shaped curve. In fact, the secondary rise in this “U”-shaped curvilinear function is considered to be a behavioral indicator for memory consolidation [18].

Holloway and Wansley [19] revisited the original study by Kamin using a passive avoidance training protocol with, however, a testing interval of 6 h for 72 h posttraining. Unexpectedly, the observed retention deficit in passive avoidance behavior appeared to be alternating, suggesting the involvement of some rhythmic biological factor(s) in the fluctuation of retention [19]. Soon thereafter, the group reported in a new study that the rhythmicity (6 h period) in retention performance reflected the inability to retrieve the newly learned experience as a result of some rhythmic internal state (state-dependent hypothesis) [20–22]. In 1975, Holloway provided additional supporting evidence consistent with the biological rhythm hypothesis, by showing that the repeated retention deficits in active and passive avoidance tasks were absent in rats whose “master circadian clock,” residing within the suprachiasmatic nucleus of the hypothalamus, was lesioned [22, 23].

### 2.1. Indirect Evidence

In recent years, the interest in the importance of the circadian system in health expanded explosively as evident by daily supporting scientific research and media coverage, linking health-related issues (physiological, psychological, and behavioral) to circadian disturbances. Thus, the saying “a healthy mind in a healthy body” has much truth to it. In regard to the importance of the integrity of the circadian system, the proverb could be further elaborated to “a healthy mind in a body with an intact circadian system.”

Many processes that influence memory, such as protein and neurotransmitter synthesis, synaptic activity, excitability, and hormone secretion, exhibit circadian oscillations [24, 25]. Thus, memory processes could be circadian-regulated through the rhythmic action of biochemical processes that influence memory formation. Holloway, after his initial work with Wansley, pursued the idea of the involvement of the circadian system in learning and memory. Holloway assumed that if an organized circadian system plays a modulation role in learning and memory, then a disruption of the organism's circadian system should affect memory processing. Accordingly, Tapp and Holloway addressed the importance of the circadian organization for normal memory processing by disrupting the circadian system of rats via phase shifting their light-dark cycle immediately after training. Rats that were trained on a one-trial passive avoidance task followed by a shift in their original light-dark cycle showed retention deficits [26]. Furthermore, the same retention deficit was also observed when rats were subjected to the same phase shift, but several days after training. The interpretation was that the amnesic effects of the phase shift were dependent on the time interval between the training and the phase shift. Tapp and Holloway also showed that the amnesic effect of circadian disorganization was not due to training and testing the animals in different circadian phases. Interestingly, the results could be divided into two clusters categorized as normal and poor retention scores. Astonishingly, the group of rats that showed poor retention performance also demonstrated abnormal reentrainment to the shifted light-dark cycles. In summary, circadian disorganization affects memory processing and supports the idea for the involvement of a circadian component in the modulation of learning and memory.

It was later demonstrated that phase shifts immediately after training or shortly before training result in retention deficits without, however, affecting more innate behaviors such as social interaction and exploration [27]. Since retention performances appear normal when testing takes place after reentrainment, the amnesic effect of circadian disorganization is more specific for memory retrieval. Fekete et al. [27] later found that the effect of the phase shift on memory retrieval during testing can be attenuated by treatment with pituitary hormones, ACTH, and vasopressin [28]. ACTH has been proposed to improve motivation and attention and increase arousal [29]. Additional evidence for the importance of an intact circadian organization in normal memory processing came from studies using a conditioned place preference task to investigate the relationship between rhythm integrity and the ability to form cognitive associations [30]. Accordingly, a consolidated circadian rhythm is a prerequisite to develop a preference for a specific context associated with a rewarding stimulus.

At the time, it was unclear whether all memory systems are influenced by the circadian system similarly. Although circadian disruption in rats also influences hippocampus-specific memory processes, the results by Devan et al. [31] suggested a consolidation view rather than a temporary memory retrieval explanation [27]. This was because animals reentrained to the new light-dark cycle for 17 days still showed retention deficits. Furthermore, a series of experiments showed that training and testing for a place navigation task in different circadian phases have no effect on the expression of this learning task. In conclusion, the different neuroanatomical substrates known to be involved in memory processing can be differentially modulated by the circadian system.

### 2.2. Direct Evidence

Using a more direct approach to show the involvement of the circadian system, Colwell's lab demonstrated that mice trained for tone, and contextual fear conditioning, learned the tasks faster during the night as compared to daytime and that memory retrieval for the acquired task was better during the day than during the night. This diurnal rhythm in acquisition and retention persisted in constant conditions (darkness). Furthermore, when animals were entrained to a reversed light-dark cycle, the rhythm in acquisition and retention also reversed [32]. Collectively, these results suggest that both acquisition and memory retrieval are circadian-modulated and provide the first direct evidence for a circadian modulation of memory processing in mammals.

Diurnal phase-dependent differences in the efficiency of memory acquisition and retention were shown in the melatonin-proficient C3H mouse strain and the melatonin-deficient C57 strain [32]. At first glance, these results were indicative that melatonin plays no significant role. However, a closer look at the profile of the acquisition curves for both strains reveals that in C3H mice, the degree of freezing was much more robust following the last training stimulus in mice trained during the day only. In C57 mice, however, significant day and night differences in the magnitude of freezing were only observed after their initial training stimulus. This suggested that elevated melatonin levels during the night might have a suppressive effect on learning the fear-conditioning paradigm and therefore may have a functional role in the rhythmic modulation of acquisition by the circadian system.

To determine whether the circadian modulation of different memory processes (acquisition, consolidation, and retrieval) is dependent on the neuronal substrate involved, mice were tested twice for two different cue-dependent fear conditioning paradigms, hippocampus-dependent (context-dependent fear conditioning) and hippocampus-independent (tone-cued conditioning). It was noted that the hippocampus-dependent conditioning and rate of extinction were more pronounced in their phase dependency as compared to the hippocampus-independent tone-cued conditioning [33]. This study along with previous findings supports the idea that different memory processes, dependent on the neuronal substrate(s) involved, are selectively regulated by the circadian system.

Importantly, choosing the right experimental parameters and conditions is essential to differentiate between phase-dependent differences in memory processes as opposed to phase-dependent differences in animal performance. A classic example is the study by Valentinuzzi et al. [34], showing that what appears at first glance as a phase-dependent difference in acquisition and retention is actually phase-dependent differences in performance, like search pattern and swimming speed as a result of daytime-dependent differences in the animal's motivation to escape from the water maze.

Is the circadian modulation of memory processing evolutionary conserved? Fernandez et al., using *Aplysia californica*, a diurnal invertebrate, showed for the first time that long-term sensitization for tail/siphon withdrawal reflex conditioning is more robust for daytime training when tested 24 h later, as compared to long-term sensitization for nighttime training [35]. Interestingly, short-term sensitization did not differ in a phase-dependent manner, which again appears to be evolutionarily conserved since studies using rodents show similar results [32, 36]. *Drosophila melanogaster* on the other hand does show a circadian modulation of short-term memory [37] and was the first model to show the involvement of the clock gene *Period* (*Per*) in long-term memory [38].

### 2.3. Where Is the Clock?

In mammals, most rhythms in physiology and behavior are downstream outputs of the circadian clock of the SCN; it would, therefore, be reasonable to speculate that the clock of the SCN is modulating memory processes. In fact, Ralph's lab investigated whether the rhythm in context learning persisted in the absence of a functional SCN clock [39] similar to the approach Holloway used when he showed that in the absence of the SCN the Kamin effect disappeared [23]. The main finding of this study is that the phase-dependent effect on learning the conditioned place preference task continued in animals lacking a functional SCN clock, suggesting the existence of an oscillator other than and independent of the SCN clock that is influencing learning. Differences in SCN dependency likely depends on the learning paradigm used. The latter is known to define the neuronal substrates involved which in turn could be influenced by distinct circadian networks. Spatial memory for instance which involves the dorsal hippocampus is again SCN-dependent [40].

This raises the intriguing question of whether the hippocampus houses a circadian oscillator and if it does, what is its function? Several studies have shown that clock gene reporter mice show rhythmic luciferase activity for the *Per2* gene in the hippocampus [41], in addition to recent findings showing that all major clockwork components of the transcriptional/translational feedback loop (TTFL) are rhythmically expressed in the hippocampus [42]. The question of what the function of the hippocampal oscillator exactly is and how it regulates hippocampal function, if at all, remains a very hot topic. We will focus on the hypothesis that suggests that clockwork components impose a modulatory function on the molecular signature involved in memory formation, thereby imposing a circadian modulation on memory processing. To short list molecular candidates targeted by cycling clockwork components requires knowledge about the key signaling and structural molecules that define the different forms of memory (short and long term) as well as the different memory processes (see Table 1).

## 3. PERIOD1 as a Modulator of Hippocampal Function

In the mouse hippocampus, long-term memory (LTM) formation is tightly coupled to de novo gene expression of plasticity-related proteins and posttranslational modifications [43–45] and relies on intact cAMP/protein kinase A (PKA)/protein kinase C (PKC)/CREB/ERK signaling [43, 46, 47] including chromatin remodeling [48–54]. Hippocampal LTM-specific cellular and molecular dynamics are clearly molded by time-of-day [55], supporting an intricate interplay between the circadian system and memory of yet unknown mechanism(s). Important components of the circadian timing mechanism and its plasticity are the members of the *Period* clock gene family (*Per1* and *Per2*) [56–60], complemented by cAMP-dependent signaling [61]. *Per1* being rhythmically expressed in the mouse hippocampus [42] and shown to modulate behavioral sensitization [62] implies a potential regulatory role for the clock gene protein PER1 in synaptic plasticity, particularly in the temporal modulation of learning and memory.

**Figure 1:**
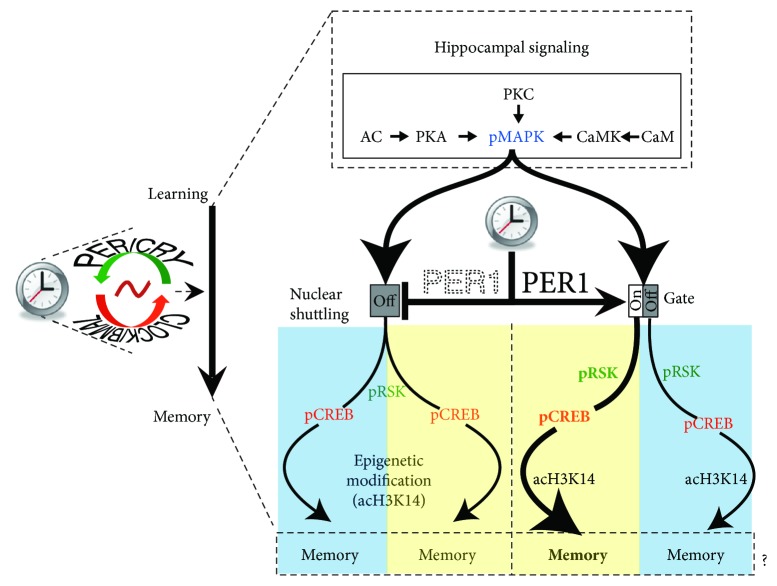
PER1, learning, and memory. Working model describing the role of the clock protein PER1 in gating daytime-dependent memory processing in the mouse hippocampus. As PER1 ties directly to memory-relevant molecular cascades, particularly pMAPK signaling, and is rhythmically expressed in the hippocampus, it can integrate circadian time into the molecular events necessary for memory processing (image modified from Rawashdeh et al. [65]).

Both lesioning the master circadian clock in the suprachiasmatic nucleus (SCN) and silencing circadian outputs blunt LTM [40, 63]. However, it is generally difficult to distinguish if these interventions affect LTM directly or indirectly by acting on endogenous hippocampal circadian oscillations via a local oscillator. Circadian core clock components are rhythmically expressed in the hippocampus of *Per1^−/−^* mice, yet their phases are shifted compared to control (*Per1^+/+^*) mice, despite having a functional SCN clock that is properly phased to ambient lighting conditions [42]. The fact that *Per1^−/−^* mice are rhythmic under both diurnal and constant conditions similar to control littermates (*Per1^+/+^*) suggests that these mice are ideal to investigate the role of PER1 in hippocampal physiology, particularly learning and memory processing as we have recently demonstrated (Figure 1) ([64–66].

There is compelling evidence that hippocampus-dependent memory is mirrored by alterations in the plasticity of LTP [45, 47] and that LTP efficiency endows a circadian component [36]. The magnitude of LTP at perforant path-granule cell synapses in the dentate gyrus (DG) is compromised in *Per1^−/−^* mice, while basic properties of synaptic transmission and presynaptic short-term plasticity appear normal, indicating that functional deficits are not likely due to alterations in network excitability [65]. Late LTP at Schaffer collateral-CA1 synapses has been shown to underlie the maintenance of LTM in living animals [44, 45]. These forms of synaptic plasticity require rapid translation of preexisting RNA in dendritic compartments [67, 68]. Collectively, the recorded reduction in the amplitude of LTP observed in *Per1^−/−^* mice may therefore suggest a specific role for PER1 in the reinforcement/consolidation of RNA synthesis-dependent LTP and associative spatial memories.

A comprehensive study demonstrated that the phosphorylation and hence the activation of both MAPK and CREB cycle rhythmically in the hippocampus [69]. Its functional significance is postulated to be important for the maintenance of long-term memories. It was later discovered that the phosphorylation of hippocampal CREB is PER1-dependent, since mice deficient for the *Per1* gene are arrhythmic in hippocampal pCREB albeit *Per1^−/−^* mice are rhythmic in their sleep/wake behavior under both diurnal and constant conditions, similar to *Per1^+/+^* mice. Furthermore, the *in vitro* induction of CREB phosphorylation via the cAMP/PKA/MAPK signaling pathway is also PER1-dependent.

Notably, long-term memory formation is dependent on different signaling cascades, many of which converge to activate the transcription factor CREB to initiate long-term memory-dependent gene expression [70–72]. The silencing of one or several of these pathways will likely alter learning-induced dynamics in CREB activation and consequently affect long-term memory formation. It has to be emphasized that while *Per1^−/−^* mice show a reduction in the amplitude of *in vivo* LTP, they do acquire long-term spatial memory; however, compared to *Per1^+/+^* mice, day/night differences in memory performance are absent (ZT02 versus ZT14). This phenomenon may be linked to the absence of day/night variations in pCREB levels in *Per1^−/−^* mice. Notably, this impairment in the temporal gating of PKA/MAPK-dependent phosphorylation of CREB in the absence of *Per1* is selective to the hippocampus, as PKA activation phosphorylates CREB in the pineal gland of *Per1^−/−^* mice [65], a model system for cAMP signaling [73].

Whether the novel findings on the role of PER1 in modulating signaling to CREB phosphorylation by regulating the nuclear translocation of the CREB kinase pMAPK-activated ribosomal S6kinase (P90RSK) *in vitro* is the mechanism for the temporal gating in hippocampus-dependent memory processing *in vivo*, is yet to be shown. This will not be an easy task as PER1 is a complex and highly regulated protein that could impose a regulatory function on hippocampal physiology via a plethora of regulatory signaling events as described next.

## 4. The Complexity Surrounding PERIOD1 Function

*Period 1*, originally named *Rigui*, is one of three homologous mammalian period genes which was first described by two independent labs in 1997 [74, 75]. The discovery of the other two orthologues (Period 2 and 3) and their interactions with core elements of the circadian oscillator creating autonomous interdependent transcriptional/translational feedback loops were published soon after [76, 77].

Within the transcriptional/translational feedback loop, the *Per1* protein (PER1) acts as a negative regulator of the clocks' transcriptional activator complex, a heterodimer consisting of the core clock protein CLOCK [78] and BMAL1/MOP3 [79]. The CLOCK/BMAL1-complex binds to E-box elements which consist of a conserved six-base-pair sequence. These E-boxes can be found in many promoter regions throughout the mammalian genome. The binding of the CLOCK/BMAL1-complex to an E-box facilitates downstream gene expression, like the period genes. Period proteins are known to cycle back to the nucleus where they bind to the CLOCK/BMAL1 complex, releasing the transcription activator complex from the E-box in the promoter region of the period genes and thus inhibiting *Per1* expression. Comprehensive views on the workings of the TTFL and its complexities can be found in the following recent reviews [80, 81].

The fact that the *Per1* promoter of both mouse and human [82, 83] consists of several E-boxes suggests a complex multimodal regulation of *Per1* gene expression, involving the circadian regulation via CLOCK/BMAL1, and other gene-regulatory elements, particularly those involved in the acute (noncircadian, immediate, ligand-mediated) inductions. One example is the CRE^∗^C/EBP element which responds to increases in intracellular cAMP levels [84, 85], while others respond to interleukin 6 [85].

In an analysis of the forskolin-induced cAMP/PKA/CREB/CRE-pathway in S49 lymphoma cells, *Per1* (mRNA) was the only clock gene transcript significantly upregulated two hours poststimulation. Such acute upregulation is similar to known immediate early genes like the inducible cAMP early repressor (ICER) [86] and less of a characteristic for genes regulating the circadian clock.

In a more recent study, the CLOCK/BMAL1-driven PER1 upregulation and the associated signal transduction pathways in human liver cells were shown to be cAMP-independent [87]. To differentiate between CLOCK/BMAL1 action and ligand-based *Per1* upregulation via second messenger-mediated pathways requires one or both pathways to be specifically inhibited using antisense techniques and/or pharmacological inhibitors [87]. It has to be noted that the dissection of and differentiation between the signaling events involved—CLOCK/BMAL1 E-box-mediated pathway versus acutely regulated second messenger-dependent pathways that are dependent on cAMP/PKA or diacylglycerol (DAG)/calcium/calmodulin-dependent protein kinase (CamK)—are rather difficult *in vivo*. It is also important to mention that *Per1* regulation can be cell- and tissue-specific, as shown in the mouse suprachiasmatic nucleus where CRE-dependent signaling appears to be essential for the circadian oscillation of clock genes [61, 88].

A recently discovered novel function for PER1 was described in primary hippocampal neurons in which PER1 signaling via the cAMP and MAPK pathways regulates the phosphorylation (activation) of its own transcription factor, CREB [65]. Here, the presence of PER1 determines the nucleocytoplasmic transport of the protein kinase pP90RSK, thereby gating downstream cAMP-dependent gene expression in the mouse hippocampus [89]. Notably, the original paper by Rawashdeh et al. utilized primary hippocampal cultures in order to study the function of PER1 in isolation of extrahippocampal influences on local hippocampal signaling [65]. This is important, because the *Per1^−/−^* mice used in this study were global knockouts.

Additional complexity in *Per1* regulation emerges from the various posttranslational modifications that PER1 can undergo. Among the first established posttranslational modifications was its phosphorylation by casein kinase 1 [90], isoforms delta and epsilon, and maybe also gamma2. However, there are many potentially alternative threonine, serine, and tyrosine residues that can be phosphorylated by a variety of protein kinases as deduced from mass spectrometry, according to PhosphoSitePlus.

Depending on the specific phosphorylation pattern, PER1 homodimers can become targets for various protein phosphatases (confirmed is PP1), as well as ubiquitin transferases [91]. Each of these phosphorylation sites can potentially be of significant importance since the mutation of only a single PER1 phosphorylation site can have profound effects on, for example, circadian feeding behavior [92]. Some of the potentially phosphorylated amino acid residues determine the cytoplasmic localization of PER1, its resistance against proteasomal degradation, and nuclear translocation [93].

PER1 interacts either directly or indirectly with PER2 and 3 and CRY1 and 2, and its nuclear translocation permits its interaction with the CLOCK/BMAL1 complex [94] as well as some additional proteins like NONO, WDR5 [57], and SFPQ [95–97]. The most recent X-ray crystallographic analysis strongly suggests that besides the known PER1-containing multiprotein complexes there are likely many others whose composition may be daytime and subcellular compartment-dependent [98].

In the near future, a combination of time-resolved immunoprecipitation or similar techniques [98, 99] in combination with mass spectrometric analysis of the different complexes, their components, and the posttranslational status of its protein components will shed new light on the complex yet delicate regulation of PER1 within a cell traveling through circadian time.

## 5. Thoughts to Consider

What is driving hippocampal rhythmicity, particularly memory relevant signaling? One possibility is that the hippocampus houses a local circadian oscillator to gate hippocampal function. This idea however is not supported on the basis of data presented by Jilg et al., which show that all core elements of the circadian clock, with the exception of *Per1*/PER1, cycle in phase [42] and not as expected for the positive components of the TTFL to be in antiphase relative to the components of the negative limb. Hence, the hippocampus although rhythmic may not contain an autonomous “ticking” oscillator. Alternatively, rhythmic input of neuronal and/or humoral origin into the hippocampus could drive day/night differences in hippocampal function. For instance, melatonin, a pineal hormone whose synthesis and release are strictly confined to only the nighttime would in principle be able to set a nighttime-specific hippocampal tone [100, 101]. Last but not least, what is the functional significance behind the diurnal and circadian rhythmicity in hippocampal signaling events? To date, much of the focus is on the genetic side of things with little emphasis on the more upstream rhythms in posttranslational modifications of kinases, their phosphatases, and phosphatase inhibitors. One could think of the diurnal and circadian variations in the activation state of signaling molecules as signatures defining different temporal states that determine the efficiency of memory processing at different times of the day and or the maintenance of long-term memories [40].

## 6. Summary and Conclusion

For an organism to register periodically, reoccurring stimuli (rewarding or harmful) require an efficient time management system to associate, retain, and recall temporal information. Molecular circadian clockworks, originally discovered in neurones of the SCN [61, 102], would be the ideal time management machinery. PER1, a core clockwork component, is also an important element in linking between circadian time cues (neuronal and/or hormonal) [40, 100] and memory processing, with cAMP signaling [61] and epigenetic modifications [103] at its center. In fact, clock genes, constituting integral parts of the cellular and biochemical machinery that store information about time, may also be a molecular prerequisite for memory storage in a much broader sense than previously anticipated. This may be relevant for adaptive brain functions in both health and disease [53, 54]. As long-term memory is afflicted in both *Per1^−/−^* mice and *Per*-mutant flies [38, 66], the memory-associated function of this clock gene appears to be preserved over some 700 million years of evolutionary distance.

## Figures and Tables

**Table 1:** Circadian modulation of memory-relevant signaling in rodents.

Signaling molecule	Short-term memory	Model organism	Long-term memory	Model organism	Memory retrieval	Model organism	Circadian/diurnal rhythmicity	Model organism
cAMP	✓	Wistar rats [104]	✓	C57BL/6 × 129/Ola mice [105]	—		✓	C57/BL6 mice [69]
PKA	X	C57BL/6J mice [47, 106] Sprague-Dawley rats [107]	✓	C57BL/6J mice [106, 108] Sprague-Dawley rats [107]	—		✓	C3H/H3N mice [64]
PKC	✓	Wistar rats [109, 110]	✓	Wistar rats [111]	✓	CD1 mice [112]	✓	C3H/HeN [64] mice
pMAPK	—		✓	C57/BL6 [113]	✓	C57BL/6 [114, 115]	✓	C57/BL6 mice [69, 116]
pCREB	X	Wistar rats [117] Long-Evans hooded rats [118]	✓	Wistar rats [117] Long-Evans hooded rats [118]	X	C57BL/6 mice [119]	✓	C3H/H3N mice [64] C57/BL6 mice [69]
AC	✓	C57BL/6 × 129/SV mice [120]	✓	C57BL/6 × 129/SV mice [120]	—		—	
CAMKIV	X	C57BL/6N mice [121]	✓	C57BL/6 mice [122]	—		✓	WKY rats [123]
CAMKII	X	129/BL6 mice [124]	✓	129/BL6 mice [124]	X	Wistar rats [125]	✓	C57BL/6 mice [116, 126]

✓: effect; X: no effect; —: unknown; cAMP: cyclic-adenosine monophosphate; PKA: protein kinase A; PKC: protein kinase C; pMAPK: phosphorylated mitogen-activated protein kinase; pCREB: phosphorylated cAMP response element-binding protein; AC: adenylyl cyclase; CAMKIV: calcium/calmodulin-dependent protein kinase type IV; CAMKII: calcium/calmodulin-dependent protein kinase type II.
